# HIV protease resistance mutations in patients receiving second-line antiretroviral therapy in Libreville, Gabon

**DOI:** 10.1186/s12879-024-09156-9

**Published:** 2024-03-14

**Authors:** Guy Francis Nzengui-Nzengui, Gaël Mourembou, Hervé M’boyis-Kamdem, Ayawa Claudine Kombila-Koumavor, Angélique Ndjoyi-Mbiguino

**Affiliations:** Département de Bactériologie- Virologie, Laboratoire National de Référence IST/VIH/Sida, Université des Sciences de la Santé, Libreville, Gabon

**Keywords:** HIV, Protease, Resistance mutations, Antiretroviral, Gabon

## Abstract

**Introduction:**

In 2022, the WHO reported that 29.8 million people around the world were living with HIV (PLHIV) and receiving antiretroviral treatment (ART), including 25‌ 375 people in Gabon (54% of all those living with HIV in the country). The literature reports a frequency of therapeutic failure with first-line antiretrovirals (ARVs) of between 20% and 82%. Unfortunately, data relating to the failure of second-line ARVs are scarce in Gabon. This study aims to determine the profiles of HIV drug resistance mutations related to protease inhibitors in Gabon.

**Methodology:**

Plasma from 84 PLHIV receiving ARVs was collected from 2019 to 2021, followed by RNA extraction, amplification, and sequencing of the protease gene. ARV resistance profiles were generated using the Stanford interpretation algorithm version 8.9-1 (https://hivdb.stanford.edu) and statistical analyses were performed using EpiInfo software version 7.2.1.0 (CDC, USA).

**Results:**

Of 84 HIV plasma samples collected from 45 men and 39 women, 342 mutations were detected. Of these, 43.3% (148/342) were associated with nucleoside reverse transcriptase inhibitors (NRTIs), 30.4% (104/342) with non-nucleoside reverse transcriptase inhibitors (NNRTIs), and 26.3% (90/342) with protease inhibitors (PIs). Most NRTI mutations were associated with thymidine analogues (TAMs) (50.7%; 75/148), including T215F/V (14.9%; 22/148), D67DN/E/G/N/T (10.1%; 15/148), M41L (9.5%; 14/148), and K70E/KN/S/R (9.5%; 14/148). Resistance mutations related to non-TAM NRTIs (33.1%; 49/148) were M184V (29.1%; 43/148), and L74I/V (8.1%; 12/148). NNRTI mutations were predominantly K103N/S (32.7%; 34/104), V108I (10.6%; 11/104), A98G (10.6%; 11/104), and P225H (9.6%; 10/104). Minor mutations associated with PIs (60.0%; 54/90) were predominantly K20I (15.6%; 14/90) and L10F/I/V (14.5%; 13/90). The major mutations associated with PIs (40.0%; 36/90) were M41L (12.2%; 11/90), I84V (6.7%; 06/90), and V82A (6.7%; 06/90). The four most prescribed therapeutic regimens were TDF + 3TC + LPV/r (20.3%; 17/84), ABC + DDI + LPV/r (17.9%; 15/84), TDF + FTC + LPV/r (11.9%; 10/84), and ABC + 3TC + LPV/r (11.9%; 10/84).

**Conclusion:**

This study revealed that HIV drug resistance mutations are common in Gabon. The major mutations associated with PIs were M41L, I84V, and V82A. There is a need for access to new NRTIs, NNRTIs, and PIs for a better therapeutic management of PLHIV in Gabon.

## Introduction

Over the past 20 years, the 51% reduction in the number of deaths due to the human immunodeficiency virus (HIV) that causes acquired immunodeficiency syndrome (AIDS) pushed these infections down to 19th place in 2019 among the world’s top causes of death since 2010 [1]. In 2022, HIV remained a major public health problem worldwide, having caused 40.4 million deaths since 1981, and its transmission continues in all countries [2]. In that year, the Joint United Nations Programme on HIV/AIDS (UNAIDS) estimated the number of people living with HIV (PLHIV) worldwide at 39.0 million, with two-thirds (25.6 million) living in sub-Saharan Africa. It also reported that, in the same year, 29.8 million people around the world were receiving antiretroviral treatment [2], that 630 000 PLHIV died of HIV-related causes, and 1.3 million new HIV infections occurred [2]. Resistance to antiretrovirals can compromise their effectiveness in treating HIV, leading to an increase in the number of infections, morbidity, and mortality associated with HIV [3, 4]. Nevertheless, access to antiretroviral therapy (ART) has improved considerably over the past decade [5]. With the ever-increasing number of PLHIV receiving antiretroviral therapy and longer treatment durations, failures of first-line treatments are increasingly being reported. Recently, 21 surveys carried on resistance linked to nevirapine (NVP) or efavirenz (EFV) showed 10% resistance among PLHIV who started first-line antiretroviral therapy. That leads healthcare workers to prescribe second-line ART treatments for PLHIV [6]. As a result, resistance to the non-nucleoside reverse transcriptase inhibitor (NNRTI) class of antiretrovirals is up to three times more frequent in people who have already been exposed to antiretrovirals [7]. The frequency of failure of first-line antiretroviral therapy for HIV infection in adults is estimated at between 20% and 82% [8]. In contrast, virological failure rates in adults on second-line regimens have been reported to range from 8 to 41% in resource-limited countries [9]. One futuristic goal that has been set is that four million patients will be receiving second-line antiretroviral treatment in sub-Saharan Africa by 2030 [10]. In 2016, WHO guidelines for HIV treatment recommended a second-line regimen consisting of two nucleoside reverse transcriptase inhibitors (NRTIs) and a ritonavir-boosted protease inhibitor (PI) [11]. The global action plan on HIV resistance to antiretroviral molecules (HIVDR) builds on the sustainable development global commitment presented in the new Agenda 2030 to end the AIDS epidemic by 2030 [12]. WHO guidelines recommend the systematic monitoring of viral load (VL) and the extension of HIV resistance testing [11, 13, 14]. For the biological monitoring of PLHIV, Gabon, through its National Programme for the Control of Sexually Transmitted Infections (PNLIST), adheres to WHO guidelines, which recommend that all HIV-positive people begin antiretroviral treatment (ART) [15]. Between 2002 and 2021, three studies on resistance mutations were carried out on patients in Gabon. First of all in Libreville in 2002, Vergne et al. reported 58% of resistance mutations [16]. Then in Franceville in 2012, Caron et al. and in 2021 Engone-Ondo et al. found 56.7% and 21.9% of resistance mutations, respectively [17–18]. However, all these studies, although focused on the protease gene, were carried out on small samples (19) or in semi-rural regions that did not cover the whole country and failed to provide a comprehensive view of the diversity of HIV antiretroviral resistance mutations related to protease inhibitors in Gabon. The aim of this study, therefore, was to investigate HIV antiretroviral resistance mutation profiles in the protease gene in PLHIV receiving second-line ARVs treatment in Gabon.

## Materials and methods

### Study type and setting

This was a cross-sectional study conducted over 24 months, from October 2019 to October 2021, at the National STI/HIV/AIDS Reference Laboratory in the Department of Bacteriology-Virology of the University of Health Sciences (USS) in Libreville, Gabon.

### Patients and ethical considerations

After approval from the Gabonese National Ethics and Research Committee (CNER) under project number PROT N°0056/2022/CNER/P/SG, 5 ml blood samples from PLHIV with virological failure (> 1000 copies/ml), were collected in EDTA tubes. Blood samples from each patient were centrifuged at 1500 rpm for ten minutes using a Rotorfix 32 A centrifuge (Hettich, Germany). The collected plasma was stored at -20 °C until molecular analyses. Signed informed consent was obtained from each patient or from legal guardians of children under the age of 11 year-old. The criteria for non-inclusion were any PLHIV who refused to participate in the study and patients whose samples were of insufficient volume. Patients were referred by clinicians from outpatient treatment centres (CTA) and infectious disease departments in Gabonese hospitals, for antiretroviral resistance mutation testing, following suspected virological failure (viral load > 1000 copies/ml), according to the WHO [19], and were recruited at their follow-up appointments. CD4 T-cell levels were also categorised according to the WHO/CDC classification into CD4 < 200/mm^3^, 200 < CD4 < 500/mm^3^ and CD4 ≥ 500/ mm^3^.

### Molecular analysis

RNA of HIV-1 was extracted from plasma using QIAamp® Viral RNA, Mini Kit (Qiagen, Courtaboeuf, France). Reverse transcriptase (RT) and protease (PROT) genes were amplified with the Invitrogen Platinum Taq DNA Polymerase Kit after retrotranscription of RNA to DNA with SuperScript III One-Step RT-PCR Kit (ThermoFisher Scientific, Waltham, MA, USA) on the Applied Biosystems GeneAmp® PCR system 9700 thermocycler. Retrotranscription of reverse transcriptase and protease genes was performed using MJ3/MJ4 and 5’PROT1/3’PROT1 sets of primers, respectively. Amplification was performed using the A35/NE135 set of primers [20]. PCR results were revealed on a 0.5% agarose gel. Positive samples were sequenced with an ABI 3730 XL DNA Analyzer [20, 21]. The sequences were analysed and edited using Chromas pro software (Technelysium Pty Ltd, South Brisbane, Australia). From the consensus sequences, resistance mutation profiles on the RT gene were generated using the Stanford interpretation algorithm version 8.9-1 (https://hivdb.stanford.edu).

### Statistical analysis

Socio-demographic data (age, sex), biological data (last CD4 T-cell count, viral load) and treatment data were collected and entered into an Excel 8.0 spreadsheet. Statistical analysis was performed using EpiInfo software version 7.2.1.0 (CDC, USA). Categorical variables were presented as frequencies. Quantitative variables were presented as adjusted odds ratios (aOR) at 95% confidence intervals (95% CI). Variables were considered to be significantly associated with outcome if the *P*-value for adjusted OR was lower than 5% (*P* < 0.05). Correlation analyses were used to identify risk factors for the occurrence of ARV resistance mutations (NRTIs and NNRTIs).

## Results

### Demographic, immunological, virological and therapeutic characteristics

A total of 84 plasma samples from PLHIV receiving ARV treatment were analysed. Forty-five of them were from male patients (53.6%) and 39 were from female patients (46.4%), giving an M/F sex ratio of 1.2 (Table 1). The median age (IQR) was 45.5 [36–52] years, with extremes of 11 and 71 years. The median age was 48 years for males and 38 years for females. The 45–60 age group was the most represented (42.9%; 36/84), followed by the 30–45 age group (35.7%; 30/84) (Table 1). In terms of T CD4 lymphocyte count, 75 samples (89.3%) had less than 200 mm^3^ (Table 1). For viral load measurements, 82 of patients (97.6%) had a virological failure (VL > 1000 copies/ml). In addition, many PLHIV had been receiving ARV treatment for 15 or 20 years (42.9%; 36/84) (Table 1).

**Table 1:** Demographic, immunological, virological, and therapeutic characteristics

Variables	N = 84	%
Gender		
Female	39	46.4
Male	45	53.6
**Age range**		
[0–15 years]	2	2.4
[15–30 years]	10	11.9
[30–45 years]	30	35.7
[45–60 years]	36	42.8
[60 to more]	6	7.1
**TCD4 lymphocytes**		
CD4 < 200 mm^3^	75	89.3
200 < CD4 < 500 mm^3^	6	7.1
CD4 ≥ 500 mm^3^	3	3.6
**Viral loads**		
VL > 1000	82	97.6
Undetectable	2	2.4
**ART duration**		
less than five years	0	0
[5–10 years]	13	15.5
[10–15 years]	30	35.7
[15–20 years]	36	42.9
[20 years or more]	5	5.9

### Therapeutic lines adopted in Gabon

In this study, all the PLHIV were on second-line ARV therapy (Table 2). Four therapeutic lines were the most prescribed: TDF + 3TC + LPV/r (20.3%; 17/84); ABC + DDI + LPV/r (17.9%; 15/84), TDF + FTC + LPV/r (11.9%; 10/84) and ABC + 3TC + LPV/r (11.9%; 10/84) (Table 2).

**Table 2:** Therapeutic lines for second-line ARVs

Therapeutic lines	Frequencies	%
TDF + 3TC + LPV/r	17	20.3
ABC + DDI + LPV/r	15	17.9
TDF + FTC + LPV/r	10	11.9
ABC + 3TC + LPV/r	10	11.9
AZT + 3TC + LPV/r	08	9.5
Others*	24	28.6
**Total**	**84**	**100.00**

ABC = Abacavir; AZT = Zidovudine; DDI = Didanosine; 3TC = Lamivudine; TDF = Tenofovir; FTC = Emtricitabine; LPV/r = Lopinavir booster with Ritonavir

*ABC + 3TC + ATV/r; AZT + 3TC + DRV/r; AZT + 3TC + IDV/r; TDF + 3TC + ATV/r; TDF + 3TC + DRV/r

### Mutation profiles observed in subjects treated with reverse transcriptase and protease inhibitors

A total of 342 mutations were detected in 84 patients. The results in Table 3 show the different resistance mutation profiles associated with NRTIs (43.3%; 148/342), NNRTIs (30.4%; 104/342) and PIs (26.3%; 90/342). NRTI-associated mutations were predominantly those associated with thymidine analogues (TAMs) (50.7%; 75/148): T215F/V (14.9%; 22/148), D67DN/E/G/N/T (10.1%; 15/148), M41L (9.5%; 14/148), and K70E/KN/S/R (9.5%; 14/148). Mutations associated with resistance to non-TAM NRTIs (30.4%; 45/148) were, respectively, M184V (29.1%; 43/148) and L74I/V (8.1%; 12/148) (Table 3). In parallel, the most frequently detected NNRTI-associated mutations were K103N/S (32.7%; 34/104), V108I (10.6%; 11/104), A98G (10.6%; 11/104), and P225H (9.6%; 10/104) (Table 3). Finally, the results in Table 3 reveal that minor mutations associated with PIs (60.0%; 54/90) were predominantly K20I (15.6%; 14/90), L10F/I/V (14.5%; 13/90), and L33F (5.6%; 5/90), compared with major mutations (40.0%) the most frequent of which were M41L (12.2%; 11/90), I84V (6.7%; 06/90) and V82A (6.7%; 06/90) (Table 3).

**Table 3:** Distribution of mutations associated with resistance to NRTIs, NNRTIs, and PIs

Codons	N = 90	%
**Major Mutations PIs**	36	40
M46I	11	12.2
I84V	6	6.7
V82A	6	6.7
I54V	4	4.4
I47V	3	3.3
L76V	3	3.3
L90M	2	2.2
I50V	1	1.1
**Minor mutations PIs**	**54**	**60**
K20I	14	15.6
L10F/I/V	13	14.5
L33F	5	5.6
T74P/S	5	5.6
F53L/Y	4	4.5
A71T/V	3	3.3
L89V	3	3.3
Q58E	2	2.2
I15V	1	1.1
I84I/M	1	1.1
K43T	1	1.1
M361	1	1.1
**Codons**	***N*** = **148**	**%**
**Mutations RNTIs**		
**Non TAMs**	**45**	**30.4**
M184V	43	29.1
L74I/V	12	8.1
**TAMs**		
K215F/V	22	14.9
D67DN/E/G/N/T	15	10.1
T219N/Q/Y	10	6.7
K65R	6	4
T69N	4	2.7
V75I	3	2
E44D	2	1.3
Y115F	2	1.3
A62V	1	0.8
**Codons**	***N*** = **104**	**%**
**Mutations NNRTIs**		
K103N/S	34	32.7
V108I	11	10.6
A98G	11	10.6
P225H	10	9.6
H221Y	6	5.8
Y188L	6	5.8
G190A	6	5.8
Y181C	5	4.8
E138A/Q	4	3.8
K101P	3	2.9
V106I	3	2.9
V197T/VE	3	2.9
M230L	1	0.9
K238T	1	0.9

### Correlations between viral load and resistance mutations

Of the 84 patients, 97.6% (82/84) had a detectable viral load. Of the 82 patients with detectable viral load, 20.7% (17/84) had major resistance mutations and 44.0% (37/84) had minor resistance mutations to protease inhibitors. However, 60.7% (51/84) only had mutations associated with nucleoside reverse transcriptase inhibitors (NRTIs) and 64.3% (54/84) only had resistance mutations associated with non-nucleoside reverse transcriptase inhibitors (NNRTIs). Furthermore, 82 (97.6%) had virological failure, and 13 (15.5%) of them had predominantly PI-resistance mutations. No statistical correlation was observed between viral load and the frequency of resistance mutations associated with protease inhibitors.

### Correlations between therapeutic lines and mutations presence

Of the four ARV combinations more widely prescribed, the ABC + DDI + LPV/r combination was correlated with the appearance of PI-resistance mutations (5.9%; 5/84); adjusted OR 2.79 to [CI95% [1.57–4.96], *P* = 0.00].

### Correlation between duration of antiretroviral treatment and presence of mutations

PLHIV who had been receiving treatment for 20 years were significantly at risk of the occurrence of PI-associated resistance mutations (2.4%; *n* = 2/84); adjusted OR greater than 9.6 at CI95% [2.93–31.46], *P* = 0.00.

## Discussion

Virological and immunological failures are problems that currently endanger the biological management of PLHIV in sub-Saharan Africa due to the emergence of resistance mutations against ARVs. The WHO therefore recommends that patients who have experienced first-line antiretroviral failure be switched to a second-line antiretroviral regimen comprising two nucleoside reverse transcriptase inhibitors (NRTIs) and a ritonavir-boosted protease inhibitor [22]. Second-line treatment failure in PLHIV is estimated at 18.8% in low-income countries [23]. The results show that the predominant sex is male (53.6%; 45/84). The median age of our study population was 45.5 years. The median age was 48 years for males and 38 years for females. For males, this is lower than in previous studies [24], while for females it is as previously reported in some African countries, although it remains lower than ages reported in Ethiopia [25–28].

In our study, 342 ARV resistance mutations were identified in 84 PLHIV. Of them, 148 (43.3%) were NRTI-associated mutations and 104 (30.4%) were NNRTIs associated mutations. These data are lower than those previously found in Zambia and Uganda [29–30]. In Zambia, 81% mutations associated with NRTIs were reported, while in Uganda, 58.8% of PLHIV have both NRTI- and NNRTI-associated mutations [29–30]. This rate was 73.2% in Suriname and 65.5% in Zambia [29, 31]. Our study showed that the emergence of drug resistance mutations is common after the failure of first-line treatment and is generally characterised by mutations affecting both NNRTIs and NRTIs, as reported between 2012 and 2018 [32–34]. The detection of NRTI resistance, in particular thymidine analogue mutations (TAMs), prior to initiation of second-line therapy should predict significantly higher odds of virological suppression [32, 33, 35].

In this study, 90 PI-associated resistance mutations were identified (26.3%), particularly two categories of patterns: major mutations in 36 (40%) patients, with the predominant codon M46I found predominantly in 11 (12.2%) PLHIV, and minor mutations in 54 (60%) patients, with the predominant codons K20I (15.5%; *n* = 14) and L10F/I/V (14%; *n* = 13). Our results were higher than those revealed in 2023 by Kiros et al., who detected codons M46I and F53L (3.9%; 2/51) in two individuals [36]. However, they were lower than those reported in 2022 in Kwazulu-Natal, South Africa, by Chimukangara et al. who found that the most frequently detected PI codons were V82A (*n* = 88), M46IL (*n* = 83), and I54MTV (*n* = 80) [27]. Furthermore, in a pooled analysis of protease inhibitor (PI)-based treatment failures in sub-Saharan Africa, 17% of patients had at least one major PI-resistance mutation at the time of treatment failure, with an association between the duration of second-line treatment and the development of PI resistance [27]. Major PI resistance was associated with a longer duration of second-line treatment [27]. This corroborates the results obtained in our study, where the patients who had received ARV treatment for 20 years had more resistance mutations. Additionally, these results were lower than those reported in 2022, in South Africa by Chimukangara et al., who found that, of the 348 samples analysed, 287 (82.5%) had at least one drug resistance mutation (DRM) and 114 (32.8%) had at least one major PI-resistance mutation [27]. Similarly, the systematic review conducted in Asia by Ross et al. in 2021 revealed that 13/39 (33%) patients had mutations associated with both NRTIs and major PI resistance [6]. These problems can be explained by the relatively limited availability of viral load monitoring in low-income countries, which favours late detection of treatment failure, leading to the development of PI mutations [37]. Protease inhibitors (PI) were lower than the figures obtained by Rossi et al. in 2021 [6].

Regarding the T CD4 lymphocyte count, patients with severe immune deficiency were in the majority, at 89.3% (75/84). These results were higher than those reported in 2019 in Ethiopia by Alene et al., which revealed 60.11% (611/1011), and in 2023 in Kenya by Ombajo et al., who reported 8.6% (34/394) of PLHIV on PIs [26, 28]. These results show the levels of immunological failure, which can be explained by immune restoration problems attributed to reconstitution of the excessive immune response following the presence of non-opportunistic infections and after the introduction of antiretrovirals [38]. For instance, the paradoxical IRIS, which manifests itself in PLHIV treated for opportunistic infections, while the clinical state continues to deteriorate after the administration of ARVs [38]. A weak thymus or defective bone marrow function could be involved in poor immune reconstitution [39]. The majority of patients were in major virological failure, at 83.3% (70/84). These results suggest that virological failure is due to viral replication of the mutated virus, caused by non-compliance, non-adherence to ARVs, or discontinuation of treatment in PLHIV. There is also a need for the country to gain access to new NRTIs, NNRTIs, and PIs, as resistance to NRTIs and NNRTIs are higher compared to PI resistance. The country currently only prescribes LPV/r as PI. It is, therefore, crucial to ask for new PI drugs such as Darunavir or other PIs to be prescribed in the event of failures of PIs, in order to manage patients appropriately. Furthermore, these results show any correlations between detectable viral load and the appearance of resistance mutations to both reverse transcriptase and protease inhibitors. The first cause is the absence of regular virological monitoring, enabling resistant mutants to be detected and molecules to change rapidly. The other cause is the appearance in patients of adverse effects specific to the molecules, and the concomitant use of drugs not prescribed by physicians (parallel supply circuit), which could be at the origin of adverse reactions. One limitation is the sequencing method used, which cannot detect resistant viral subpopulations that are lower than 20%, underlining the call for NGS for such studies, for better patient management.

The therapeutic lines TDF + 3TC + LPV/r (20.3%; 17/84); ABC + DDI + LPV/r (17.9%; 15/84), TDF + FTC + LPV/r (11.9%; 10/84), and ABC + 3TC + LPV/r (11.9%; 10/84) were the most widely administered. These results are lower than those obtained in the study conducted in South Africa, which reported that the most prescribed second-lines were AZT + 3TC + LPV/r (47.1%; 164/348), TDF + 3TC + LPV/r (25.6%; 89/348), and ABC + 3TC + LPV/r (13.2%; 46/348). Furthermore, in Ethiopia in 2019, Alene et al. reported that the rate of treatment failure was higher for patients receiving second-line treatment with TDF-3TC-LPV/r (34.72%; 351/1011) and AZT-3TC-LPV/r (10.48%; 106/1011), compared to patients receiving ABC-DDI-LPV/r (18.69%; 189/1011) regimens [28].

## Conclusion

This study revealed that HIV drug resistance mutations are common in Gabon. The major mutations associated with PIs were M41L, I84V and V82A. There is a need for access to new NRTIs, NNRTIs, and PIs for better therapeutic management of PLHIV in Gabon.

## Data Availability

All data can be found from the corresponding author upon request.

## Abbreviations

3TC: Lamivudine
ABC: Abacavir
ARVs: Antiretrovirals
AZT: Zidovudine
DDI: Didanosine
EFV: Efavirenz
FTC: Emtricitabine
NVP: Nevirapine
IRIS: Immune Reconstitution Inflammatory Syndrome
LPV/r: Lopinavir boosted with Ritonavir
CHUL: Centre Hospitalier Universitaire de Libreville
CHUMEJE: Centre Hospitalier Universitaire Mère et Enfants Jeanne Ebori
CHUO: Centre Hospitalier Universitaire d’Owendo
CTA: Centre de traitement ambulatoire (outpatient treatment centre)
HIAA: Hôpital des Instructions des Armées d’Akanda
HIAOBO: Hôpital des Instructions des Armées Omar Bongo Ondimba
NRTIs: Nucleoside Reverse Transcriptase Inhibitors
NNRTIs: Non-Nucleoside Reverse Transcriptase Inhibitors
PI: protease inhibitor
PLHIV: People living with HIV
VL: Viral Load
